# Experimental and Numerical Studies of “Wood–Composite” Reinforcement in Bending Sheared Wooden Beams Using Pre-Stressed Natural and Artificial Fibers

**DOI:** 10.3390/ma18184418

**Published:** 2025-09-22

**Authors:** Agnieszka Katarzyna Wdowiak-Postulak, Grzegorz Świt, Aleksandra Krampikowska, Luong Minh Chinh

**Affiliations:** 1 Department of Materials Strength and Structural Diagnostics, Faculty of Civil Engineering and Architecture, Kielce University of Technology, al. Tysiąclecia Państwa Polskiego 7, 25-314 Kielce, Poland; gswiter@gmail.com (G.Ś.); akramp@tu.kielce.pl (A.K.); 2 Department of Transportation Engineering, Faculty Civil Engineering, Thuyloi University, Hanoi 116705, Vietnam; chinhlm@tlu.edu.vn

**Keywords:** glued laminated beams, structures made of wood and wood-based materials, shear bending, reinforcement, pre-stressing, natural and man-made bars, FEM

## Abstract

Recent studies have confirmed the effectiveness of using natural fibers and fiber-reinforced polymer (FRP) composites as methods to improve the mechanical properties of timber structures. This improvement is particularly evident in static and dynamic flexural and shear performance. Moreover, there is a paucity of literature pertaining to numerical models that predict the non-linear behaviour of low-quality timber beams reinforced with natural and man-made fibers. The present article expounds upon a shear bending study of timber beams reinforced with bars in addition to other materials. The experimental study yielded the following findings: the best properties were obtained with hybrid reinforcement, in comparison to the reference beams. The enhancement of load-bearing capacity and stiffness for beams that have been reinforced with pre-stressed basalt bars was found to be the most advantageous, with increases of approximately 17% and 8%, respectively. Natural fibers exhibited slightly lower values, with an increase in load-bearing capacity and stiffness of approximately 14% and 3%, respectively, when compared to beams that had not been reinforced. Moreover, the numerical analyses yielded analogous results to those obtained from the experimental study. The numerical models thus proved to be a valid tool with which to study the influence of the reinforcement factor.

## 1. Introduction

Despite the protective measures taken by manufacturers, wood is characterized by limited durability and high susceptibility to biological corrosion. Wood and wood-based materials also have low resistance to moisture, which weakens their internal structures and reduces their strength. Therefore, these factors confirm the need for the proper, systematic and professional maintenance of wooden buildings [1]. Technical inspections are important in this regard, as they enable the early detection of destructive processes and their effects, as well as assessing suitability for further use, determining the causes of damage, and identifying ways to strengthen the structure [1].

Currently, the number of studies on the use of composites to reinforce wooden elements in medium and high-rise buildings is increasing [2,3,4,5]. Many composite systems use engineered timber products such as laminated veneer lumber (LVL), laminated timber, and cross-laminated timber (CLT), primarily because they have better mechanical properties and are more reliable than sawn solid timber [2,3,4,5,6]. Buildings such as Oakwood Tower and River Beech Tower demonstrate the need for more reliable columns and construction beams [2,6]. Improved load-bearing capacity and stiffness are also important in high-rise construction, as they prevent lateral displacement and enable the design of rigid connections [2,4,6]. Sanner [6] provides an overview of work on material modifications, including the hybridization of wood with steel or fiber-reinforced polymers (FRP), among others. Glued-in rods, FRP reinforcements, or steel rods embedded in wood provide an easy, concealed way to assemble components with improved corrosion and fire resistance compared to wood fasteners, such as those found in studs [6,7,8,9]. The same is true for timber floors, where a series of parallel support beams made of timber support a single layer of timber planks, or where infill is placed beneath the pavement. These usually do not meet current load-bearing capacity and stiffness requirements, so structural reinforcement is necessary. For existing floors, an appropriate retrofitting technique must be chosen. New materials (e.g., timber, concrete, steel or FRP) are often added to the upper or lower surface of the beams [7,8]. Based on the authors’ research, it was found that the most common technique for strengthening timber floors is to connect the compression slab to the beams using steel connectors (timber–concrete connection). Other solutions involve adding external steel or FRP plates (bonded or installed close to the surface) to the timber beams [9,10,11,12,13,14,15,16,17]. These reinforcements are appropriate for the underside of the beams, effectively increasing the load-bearing capacity while having little effect on stiffness [7,18]. This study [19] experimentally investigated the influence of EDSTC characteristics under different loading regimes, aspect ratios, and FRP thicknesses. It was found that the EDSTC exhibited favorable axial properties under different loading regimes and that the circumferential strain of the FRP in cyclically loaded columns was reduced by 10%.

However, the important element here is the method of reinforcement, the most innovative of which in recent times is the use of FRP materials. It should be remembered that the concept of using FRP as a reinforcing material involves using one or more FRP elements as unidirectional or multidirectional fiber arrangements to achieve a correct “wood–composite” bond. The correct orientation of the FRP fibers will then ensure the reinforcement has satisfactory mechanical properties as an integral structural element after resin impregnation and curing. FRP has a number of advantages such as high strength-to-weight ratio, excellent durability, corrosion resistance, ease of application, low transportation cost, and accelerated assembly process [20]. Glass fiber-reinforced polymer (GFRP) has low electrical and thermal conductivity and low manufacturing costs compared to other FRP materials such as carbon fiber-reinforced polymer (CFRP), among others [20]. GFRP and BFRP, on the other hand, are associated with perhaps a higher initial material cost compared to equivalent steel structures, but they have low maintenance costs over the life of the structure [20]. Basalt fibers have high fire resistance, sound insulation, and internal vibration damping capacity, as well as high fatigue strength and corrosion resistance. Glass fibers, on the other hand, are characterized by high mechanical strength, considerable flexibility, corrosion and weather resistance, and good thermal and electrical insulation. Being derived from the jute plant, jute fibers are characterized by being biodegradable and environmentally friendly; they have high tensile strength, good air permeability, and have insulating and anti-static properties, but they can also be susceptible to water damage. In summary, fiber-reinforced polymer (FRP) composite materials have very good stiffness and strength, and they have found application in the aerospace, automotive, and construction industries [21]. Steel and composite materials, on the other hand, are now widely used in the reinforcement of timber elements in many fields; in the study of carbon fiber polymer rods (CFRPs, BFRPs, GFRPs); and as tapes, mats, and steel rods for the reinforcement of glued beams [9,10,11,12,13,14], [22]. It should be noted that steel is increasingly being replaced by composite materials due to their light weight, high strength, and corrosion resistance. In the work of Dewey et al., the authors used CFRP and GFRP bars to reinforce deteriorated timber bridge beams. Their studies showed that the load capacity and stiffness of the repaired elements improved significantly [23]. Bergner et al. used carbon fiber and basalt fiber to reinforce spruce timber specimens, while two methods (vacuum infusion and TowPregs) were specified for composite fabrication, [24]. However, Qin’s paper showed a study on the use of GFRP to reinforce jointed timber beams with lap joints, and the flexural and shear strengths of the reinforced beams were determined [25,26]. In this study [27], composite sheets made of local natural fibers (jute fibers) were investigated in the shear strengthening of reinforced concrete structures. The reinforcement increased the shear strength of RC beams by 28–131% using the strip wrapping technique and by 140–175% using the full-length wrapping technique. Subsequently, the paper [28] presents tests to determine the maximum performance of glulam beams glued together with BFRP bars. Design joints of two rows of two bars per cross-section with 10 mm bars showed the best efficiency in terms of moment capacity and maximum displacement in the failure phase. A subsequent study [29] determined the post-peak performance of recycled aggregate concrete reinforced with steel microfibers using in situ 4D computed tomography and digital volume correlation with fiber doses of 1.0%, 2.0%, and 2.5%. The results showed that steel microfibers increased the strength and ductility of concrete, reducing crack volume by 12.3% and crack width by 28.7%. Fiber bridging controlled crack propagation and stress redistribution while revealing concentrated strains at fiber aggregation points. In a subsequent work [30], high-resolution 4D in situ computed tomography reconstruction technology was used to investigate the effect of fiber arrangement on the mechanical properties and optimization of the transition zone in high-strength carbonized aggregate recycled concrete, and it was found that fiber arrangement significantly affects the mechanical properties of high-strength carbonized aggregate recycled concrete, especially the peak stress, elastic modulus and post-peak elastic modulus. Moreover, the fiber arrangement improves the transition zone, and the optimized fiber angle distribution effectively reduces the width of the transition zone, thereby increasing the durability and strength of the concrete.

Such reinforcement also makes renovating historic buildings convenient. Renovating ancient wooden structures preserves the building’s originality while allowing for the partial replacement of elements using different connections, such as wood–composite and overlapping connections [21]. In their work, Li et al. used self-tapping wood screws [31]. Jensen et al. investigated the shear strength of beams with glued-in rods [32]. The use of steel plates or FRPs is common in the rehabilitation of timber structures, particularly beams and power poles [2,20]. The load-bearing capacity, stiffness, and ductility of timber columns have been improved by testing them with FRP [33] and steel profiles [34], improving load-bearing capacity, stiffness and ductility. Researchers Hu and Gao [35] performed a test with an H-shaped steel profile encased in glued Douglas fir (*Pseudotsuga menziesii*) wood bonded with resorcinol glue and two-component epoxy resin. After the test, the composite posts (1.100 mm long) showed a 52% increase in load-bearing capacity and a 197% improvement in stiffness, and the posts (2300 mm long) both increased in stiffness and load-bearing capacity by 43%. The work by Kia and Valipour [36] investigated low-quality forms of timber, i.e., pine (*Pinus radiata*) (grade MGP10) and Douglas fir (*Pseudotsuga menziesii*) (grades F5 and F7), with embedded steel bars (yield strength 300–660 MPa). Low-cost coniferous timber with small cross-sections was used for the tests. Reinforcing the structures with two-component epoxy glue and screw fixings resulted in the structures showing an increase of up to 103% in load capacity compared to unreinforced specimens [36]. The next paper presented [37] a study of wooden beams reinforced with fiber-reinforced polymers (FRP) using numerical modeling (FEM) with Simulia ABAQUS software, taking into account the anisotropic nature of the wooden materials and the non-linear behaviour of both solid wooden beams and wooden beams reinforced with FRP fibers. The numerical results of the load–deflection curves were found to be in close agreement with the experimental results. The next study [38] involved conducting experimental studies to investigate the adhesion characteristics and performance of NSM basalt FRP reinforcement with solid wood structures. The average load-bearing capacity of the adhesive-reinforced NSM FRP elements was 16% higher than that of the corresponding unreinforced beams. Another study [39] presented a modelling approach to predict the behaviour of wooden beams reinforced with carbon fiber-reinforced polymer (CFRP) composites based on the orthotropic constitutive properties of the wood species. An optimal CFRP ratio was obtained, beyond which no further increase in strength was achieved.

The analysis of reinforcing timber beams with fiber composites can also encounter some problems. Among these, the most important ones (as described above in the literature) are those related to the selection of the correct composite material, the reinforcement application technique, and, above all, the interaction with the wood and its defects. Therefore, the most important issue is the interaction between the wood and reinforcement and its effect on defects, which is why experimental, theoretical, and numerical analyses are presented below. However, the disadvantages of these materials are the high price of CFRP and GFRP laminates and the corrosion of steel components. Natural fiber-based composite reinforcements may therefore be an innovative and future-proof alternative for reinforcing timber structures. Furthermore, there is a lack of experimental, theoretical, and numerical studies on the interaction of reinforcement with timber as presented in this article. Very few such studies have been conducted on beams made from structural lumber of different quality classes using both man-made and natural fibers. Consequently, this research could represent the future of sustainable, low-cost, environmentally friendly construction with significant strength, particularly when combined with wood.

**Figure 1:**
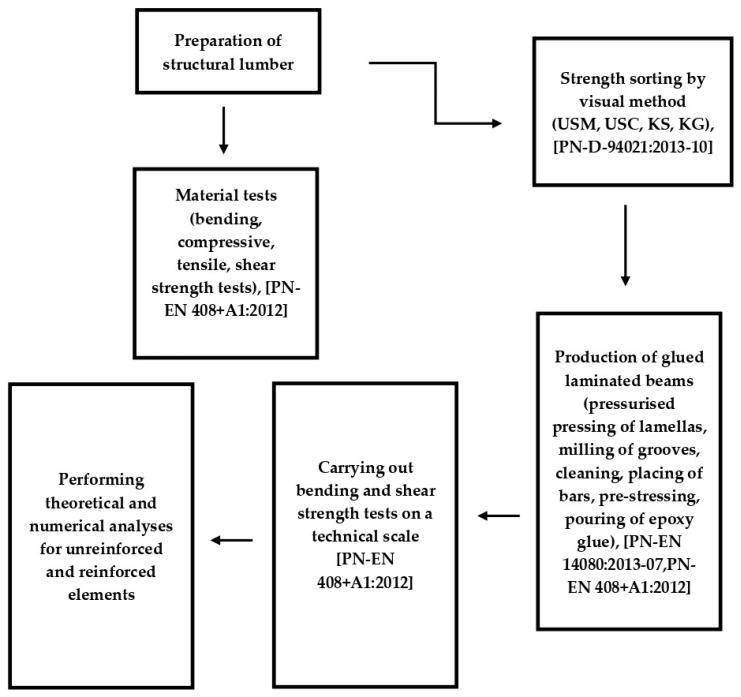
Stages of experimental, theoretical, and numerical research [40,41,42].

This paper presents the results of experimental, theoretical, and numerical analyses carried out on beams reinforced with pre-stressed composite and artificial bars. The numerical model was analysed using experimental data. The numerical results were used to analyse the static performance of the “wood–composite” reinforcement. The use of glued-in rods is a relatively new option for effectively connecting wooden structural elements. It should be noted that most wooden buildings are single-story residential buildings, which can be an effective solution for the renovation of aging wooden beams. This article presents an experimental, theoretical, and numerical program related to the evaluation of the performance of beams made of glued laminated timber and wood-based materials using glued-in rods made of basalt, glass, and jute. The same reinforcement configuration and rod diameter were specifically used. Three repetitions were tested for each type of reinforcement, and a four-point bending–shear configuration was adopted to determine the load-bearing capacity, stiffness, and deformation of the wood and reinforcement, as well as the wood–glued-in rod connection. The stages of experimental, theoretical, and numerical research are shown in Figure 1.

## 2. Materials and Methods

### 2.1. Timber with Epoxy Glue Reinforcement (Basalt, Glass, Jute)

The structural sawn timber was *Scots pine* (*Pinus sylvestris* L.) sourced from the Lesser Poland Natural Forest Region and from the beginning and end of the growing season. The kinds of timber were visually sorted by carefully viewing all pieces according to PN-D-94021:2013-10 [40]. During the study, the structural and geometric characteristics of the tested wooden samples were analysed and classified into specific strength classes using a visual method. Additionally, the average grain width, moisture content, mass, and density of the wood samples were measured in air-dried conditions. During the visual sorting, the defects and structural and geometric characteristics of the wood were determined. These included knots, cracks, graininess, fiber twists, the presence of rot and insect pavement, blue stain, heartwood, density, processing, and shape defects (oblongs, curvature, wiry, cracks, wavy grains, irregularity of planes and sides, and non-perpendicularity of faces). During sorting, the weakest cross-section with the highest concentration of defects (e.g., knots) was found in the assessed piece of lumber. The strength of the weakest cross-section determines the strength of the entire piece of lumber. By examining the weakest cross-section, the lumber strength is determined over its entire length either by assigning it an appropriate grade or by declaring it a reject. The primary feature indicating the strength class of the lumber is the knots. When analysing knots, the worst cross-section is considered, regardless of its position from the face of the lumber. For the indicated knotted cross-section, the knot ratios are determined by plotting them closer to the face of the lumber. By comparing the area of knots present to the total area of the cross-section of the lumber, the overall knottiness index (USC) is determined. The overall knottiness index (USM) is determined by comparing the area occupied by the knots to the total area of the lumber’s cross-section. The marginal knot index (USM) is determined by comparing the area occupied by knots located in the marginal zone to the cross-sectional area of this zone (see Table 1). Coniferous structural lumber, intended for beams, was divided into quality classes: KS (medium-quality class) and KG (lower-quality class). In addition, all material parameters of the structural lumber were obtained by conducting material tests in accordance with EN 408 [41], (see Table 2 and Table 3). The modulus of elasticity or bending strength testing was carried out by 4-point bending, and the compressive strength was obtained through experimental compression tests. Additionally, tests were carried out on small samples, and the average values for 4 lamellas of structural sawn timber in glued beams are given in Table 4. Additionally, material tests were carried out on small bending samples with dimensions of 20 × 20 × 400 mm, 30 × 30 × 600 mm, 20 × 20 × 120 mm, and 30 × 30 × 180 mm in accordance with the PN-EN 408+A1:2012 standard [41]. The results of the characteristic density, bending strength parallel to the fibers, and compressive strength parallel to the fibers are shown in Table 4.

The properties of the rods and epoxy adhesive are shown in Table 5 and Table 6.

The material parameters were tested or provided by the manufacturers. The bonding agent was S&P Resin 55 HP, a two-component, solvent-free epoxy resin-based adhesive with an amine hardener [43]. This glue was used to connect glued laminated beams and bars because of its liquid consistency, which allows the epoxy glue to better penetrate the wood pores and the bars’ ribs.

According to the manufacturer’s data sheet [43],

- it is solvent-free and free of volatile components;
- after hardening, it has high strength parameters;
- it is resistant to alkalis, diluted acids, salt solutions, mineral oils, aliphatic hydrocarbons, and atmospheric conditions;
- it cures without shrinkage.

Ribbed, pre-stressed bars made of basalt, glass and jute with a round cross-section and a diameter of 10 mm were used for reinforcement. The density of the composite rods was approximately in the range of 1.9 ÷ 2.10 g/cm^3^, and the density of the jute rod was approximately 1.2 g/cm^3^. The material specifications after tensile testing are shown in Table 6. Basalt and glass fibers were used due to their lower price-to-strength ratio compared to other artificial fibers. They were selected for their strength, biodegradability, durability, and breathability. They also offer excellent tensile strength, abrasion resistance, water absorption, and quick-drying properties. Glass and basalt composite rods have more than twice the tensile strength of steel rods; they are resistant to corrosion, acids and alkalis; they are four times lighter and twice as durable; and they are lower in price. The advantage of glass fibers, however, is their excellent wettability with polymers, which means resin adheres easily to them, minimizing the number of air voids at the interface between these materials. This results in high adhesion between the fibers and the surrounding polymer material, increasing the adhesive forces between them and ensuring better cooperation. They are characterized by high tensile strength, lack of electrical conductivity, and low thermal expansion. Basalt fibers have better physical and mechanical properties than glass fibers and are also relatively inexpensive. Therefore, they are the most commonly used raw materials for the production of composite rods. Basalt fibers are characterized by high fire resistance, excellent acoustic insulation, high fatigue strength, high hardness, and corrosion resistance. Jute fibers have high tensile strength and are stiff and resistant to mechanical damage but have low elasticity and extensibility.

### 2.2. Preparation of Research Elements

**Figure 2:**
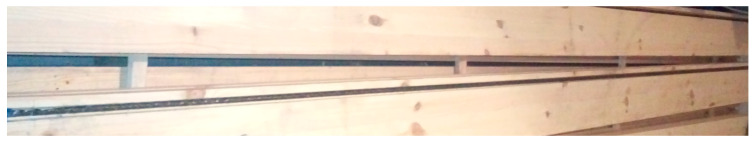
Preparation of reinforcement for glued laminated timber beams.

The bending-shear test beams were divided into four groups (see Figure 2). Twelve flexural and shear beams (beam designation A1 to A3, B1 to B3, S1 to S3, and J1 to J3) of class GL 24c according to PN-EN 14080:2013-07 [42] were prepared and tested on the basis of PN-EN 408+A1:2012 [41], (Table 7). Type A beams (A1, A2, and A3) were unreinforced beams. Experimental investigations were carried out to determine the laminated timber beams reinforced with a pre-stressed system of artificial and natural fiber rods (Figure 2). All the geometric characteristics of the 12 full-size glulam beams, made of pine, are shown in Table 7.

Pre-stressing of the composite bars was achieved at 15 MPa (5 mm thick plates, nuts). In the study, the choice of dimensions of the pre-stressing elements was dictated by the authors’ intention to prevent damage during experimental testing and to obtain a high ‘wood–composite’ adhesion. On the basis of preliminary research, suitable composite materials and the dimensions of the elements required for pre-stressing the timber beams were discerned and selected. Pre-stressing of the composite bars was achieved at 15 MPa (5 mm thick plates, nuts).

**Figure 3:**
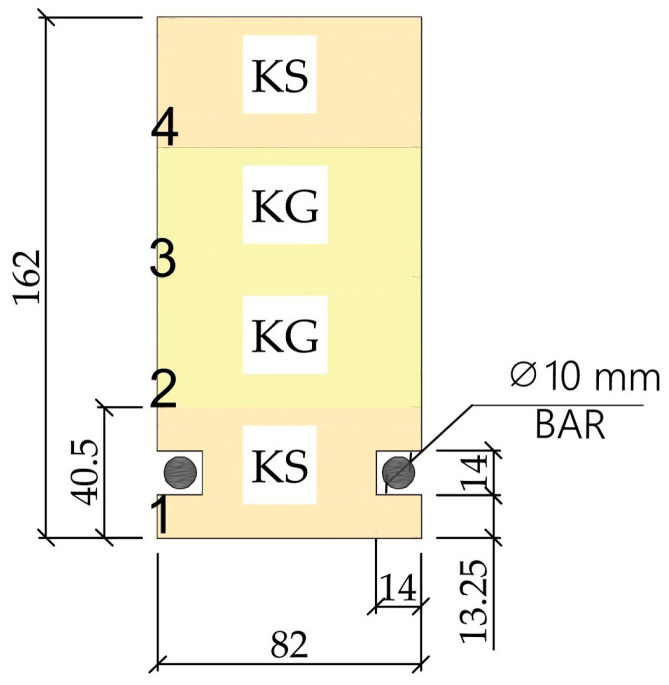
Schematic diagram of reinforcement of glued laminated beams [dimensions shown in mm], [Lamella 1, Lamella 2, Lamella 3, Lamella 4].

Figure 3 shows the “wood–composite” strengthening scheme of the beams. The beams were reinforced using basalt, glass, and jute rods located in the tensile zone of the beams. Two grooves 14 mm deep and 14 mm wide and long along the length of the beams were cut along the bottom lamella symmetrically on both sides of the beams. The dimensions of the grooves were chosen because a bar diameter of 10 mm was used and lagged with 2 mm epoxy glue to achieve the best adhesion of the reinforcement to the wood. Two FRP and jute rods 10 mm in diameter were glued to the grooves using S&P Resin 55 HP epoxy adhesive (S&P Polska Sp. z o.o., Malbork, Poland). The rods extended 100 mm beyond the area of the beams in order to correctly assemble the wood–composite reinforcement and obtain correct adhesion during the experimental tests. In addition, a layer of BFRP matting was wrapped around the bars at the ends of the beams and near the ends of the FRP bars. The support zone elements at the anchorage point were wrapped with bidirectional layers of 0°/90° woven roving, and their cross-strands improved the anchor strength. The bonding surface of the beam and bars was smoothed with sandpaper before application of the adhesive, bars, and mats.

### 2.3. Experimental Studies

There were 3 comparison beams (A1, A2 and A3) initially tested as control beams without reinforcement and without the introduction of pre-stressed bars. In contrast, the other beam elements were reinforced with pre-stressed bars.

Experimental investigations were carried out to determine the static work of the beams reinforced with the pre-stressed system and to assess the effectiveness of the “wood–composite” reinforcement.

**Figure 4:**
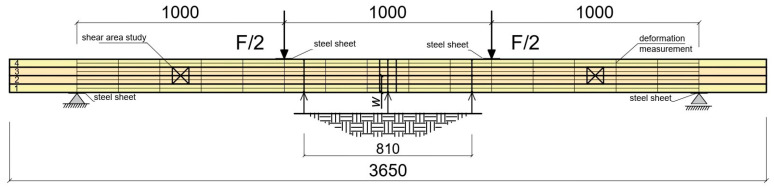
Schematic diagram of the experimental bending–shear tests [dimensions shown in mm], [Lamella 1, Lamella 2, Lamella 3, Lamella 4].

**Figure 5:**
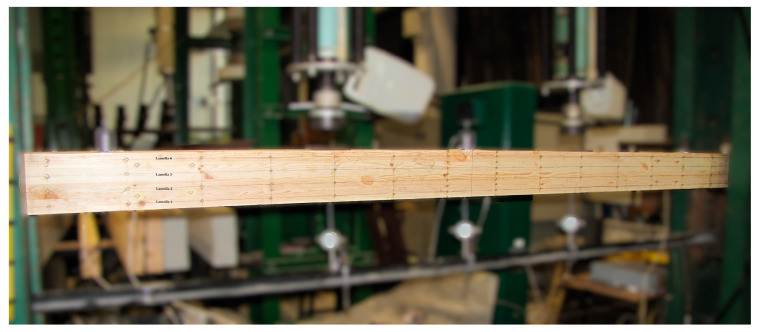
Strength testing of beams in bending and shear.

**Figure 6:**
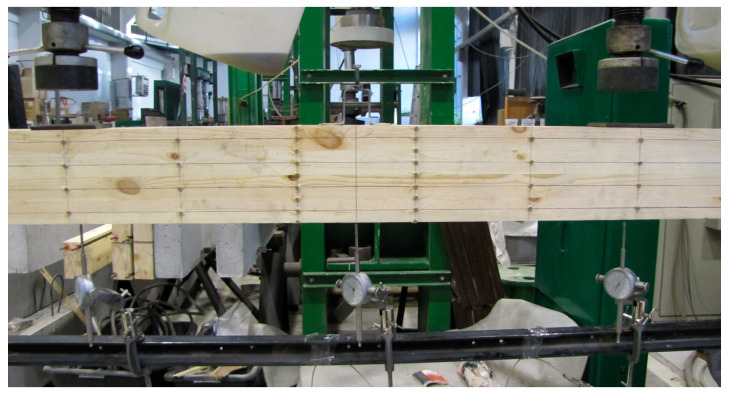
Arrangement of sensors for measuring deflections.

Experimental tests were carried out using four-point shear bending (Figure 4, Figure 5 and Figure 6). Vertical deflection was measured using dial gauges. Loads were applied with two point-concentrated forces in the form of two actuators with a piston area of 50 cm^2^ and a maximum exerted pressure of 10 MPa from VEB Werkstoffprufmaschinen Leipzig (Leipzig, Germany), (Figure 5). The load was tested with an accuracy of 0.1 kN, and deflection was measured with an accuracy of ±0.1 mm. During the experimental test, the value of the loading force and the displacement of the beam at the centre of the span and over a length of 5 h were recorded for the different loading levels, and the deformations of the wood, man-made fiber and natural fiber composites were measured using a fixed-base mechanical extensometer of the ‘Demeck’ type (Figure 6). The mechanical sensors for measuring deflections were removed before the beams were destroyed due to the possibility of damage.

All beam types tested were pinned–supported–non-sliding beams with a span of 3.65 m. The span length of the four-point shear bending system was 3.0 m. The beams were supported on a testing machine using a hall plate at each end above the support, which was 50 mm wide, 250 mm long, and 5 mm thick. Point loads were transferred from two actuators and applied to the beams by other steel plates.

### 2.4. Theoretical and Numerical Studies

The aim of the theoretical and numerical analysis was to investigate the structural behaviour of wood reinforcement with natural and artificial fibers and the static work of the “wood–composite”. In connection with determining the effectiveness of the numerical model of the reinforced beams, the results of the numerical analysis were compared with the experimental results.

Wood was considered an orthotropic material with independent mechanical properties in three mutually perpendicular directions—the longitudinal axis is parallel to the grain, the radial axis is perpendicular to the grain (in the radial direction), and the tangential axis is perpendicular to the grain (in the tangential direction)—showing the behavior of wood under the influence of different values of stresses and strains.

The numerical model was created using ANSYS 16.0 software and the Static Structural module for the construction of a finite element model (FEM).

The modeling steps are as follows.

**Figure 7:**
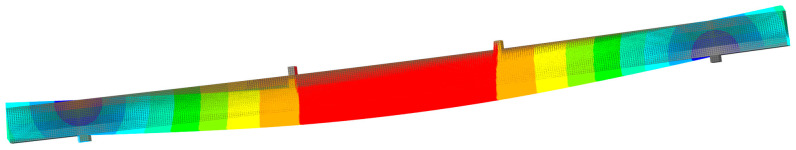
Example image of a model created in ANSYS.

- Representation of the geometric properties of the wood, reinforcement, and adhesive joint: Geometric models of the beams were created in CATIA V5, consisting of blocks serving as supports and points of application of loading forces, lamellas, bars, and the adhesive filling the space between the lamellas and bars.
- Determination of the material properties of the wood, fibers, and epoxy adhesive (including the modulus of elasticity and Poisson’s ratio) in three orthogonal directions: The mechanical properties of the wood, epoxy adhesive, basalt fibers, glass fibers, and jute given in Table 8 were derived from experimental studies (Table 1, Table 2, Table 3, Table 4, Table 5 and Table 6) or literature references [37,44,45]. Adjustments were made and applied to the material model or parameters, establishing constraints, interactions, and boundary conditions. The remaining data were automatically calculated in the ANSYS environment.
- Generation of the appropriate mesh: The finite element mesh consisted of hexagonal and tetragonal elements. Hexagonal elements with a dimension of 10 mm were used for the lamella and support geometries. Tetragonal elements with a dimension of 5 mm were used for the bars and the adhesive area surrounding them. The KS and KG structural lumber and supports were modelled as hexagonal elements with a dimension equal to 10 mm. The rods and epoxy glue were defined as tetragonal elements with a dimension equal to 5 mm.
- The configuration settings in ANSYS were obtained by defining the appropriate analysis type and specifying additional parameters.
- Analysis and monitoring of the results were conducted to obtain the results of the modeling procedure, and comparative results were obtained from the experimental methods (see Figure 7).

**Figure 8:**
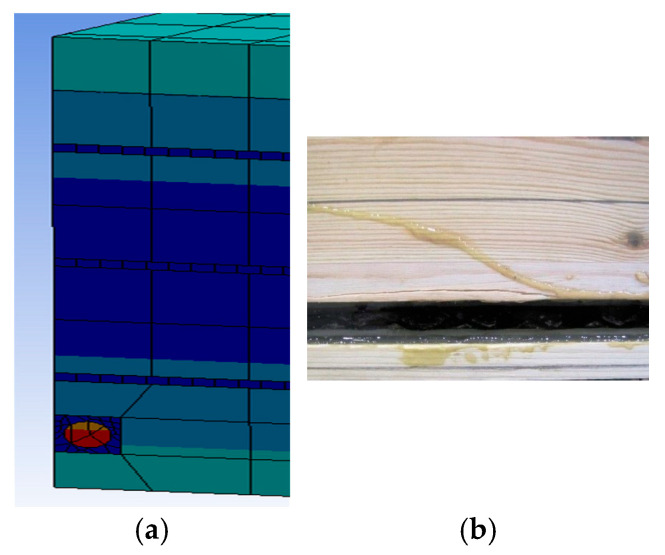
Finite element model mesh of wood–composite reinforcement: (**a**) FEM; (**b**) experimental reinforcement of beam B1.

The timber elements and rods were modelled as linear elastic–orthotropic materials. The boundary conditions of the ends of the beams followed the actual work of the experimental tests. The connections between the lamellas were modelled using a bonded connection (see Figure 8).

## 3. Results

### 3.1. Deflection Analysis of All Beams

**Figure 9:**
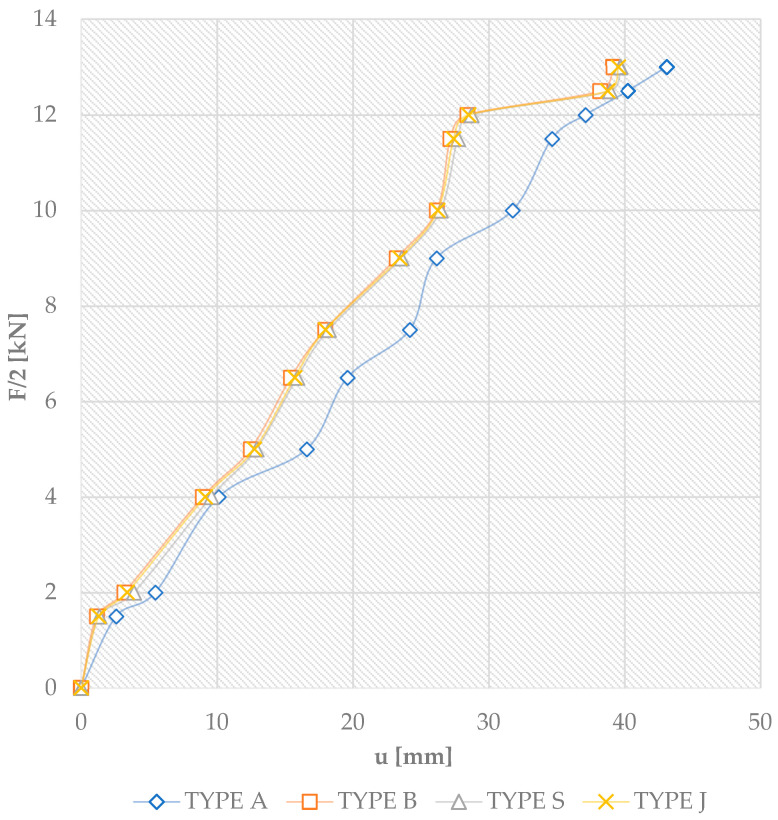
A graph of the relationship ‘F/2-u’ for all beams glued in layers at the mid-span [u—deflection].

Figure 9 shows the F/2 load [kN]–deflection curves for four-point shear bending. These are the mid-span load and vertical displacement curves for all tests. Each graph identifies the results of all beam types, with Type A being the average values for the three beams A1, A2, and A3. The others are reinforced beams: Type B-3 are beams reinforced with pre-stressed basalt bars; Type S-3 are beams reinforced with pre-stressed glass bars; and Type J-3 are beams reinforced with pre-stressed jute bars.

The average ultimate load for unreinforced Type A beams was 35 kN with a corresponding deflection of 49.2 mm. In the case of unreinforced beams, failure usually occurred abruptly due to the inhomogeneity of the elements and the wood defects present in the tension zone. Then, the laths of the structural lumber suddenly made a loud noise and cracked in the tension zone in the area of the knot present. Obviously, prior to failure, the load–deflection or strain curves at section height were close to the line graph. In the case of beams glued in layers with pre-stressed basalt bars, i.e., beam Type B, the mean value for the ultimate load was 41 kN with a corresponding deflection of 45.5 mm. In the case of basalt reinforcement, the bars ideally compensate for the non-uniform structure of the timber. In contrast, beams using pre-stressed glass bars were 37 kN, 48.8 mm; pre-stressed jute bars were 40 kN, 47.9 mm. During testing, jute fiber showed similar elasticity to glass and achieved a similar load-bearing capacity to basalt, although it had a lower modulus of elasticity due to the better cooperation of the wood–composite–glue joint as a result of the increasing loading forces and the correct cooperation after the application of additional pre-stressing elements (and thus better pre-stress efficiency). The deflections for all the beams tested, as shown in Figure 9, were approximately linear up to their ultimate loads. Figure 9 does not show the course of the line after the failure of the elements. From the tests, it was concluded that very good adhesion of the “wood–composite” joint was achieved. Due to the proper anchoring and pre-stressing during the tests (and the sudden cracking in the area of knots), the deflection of the beam increased; the load and deformation decreased; and the combination of ribbed bars and wood prevented crack propagation. The reason for this was the significant bond strength between the bars and the timber. Cracks appeared in beams S2 and J3 between the bars and the wood, but only after the rapid splitting of the fibers in the compression zone and the shearing of the fibers along lamella 2. The compressive destruction of the wood on the upper surface appeared first at a load of about 24.0 kN, and shearing occurred rapidly in beams B2, J1, and J3.

### 3.2. Wood Deformation and Reinforcement Analysis of All Beams

**Figure 10:**
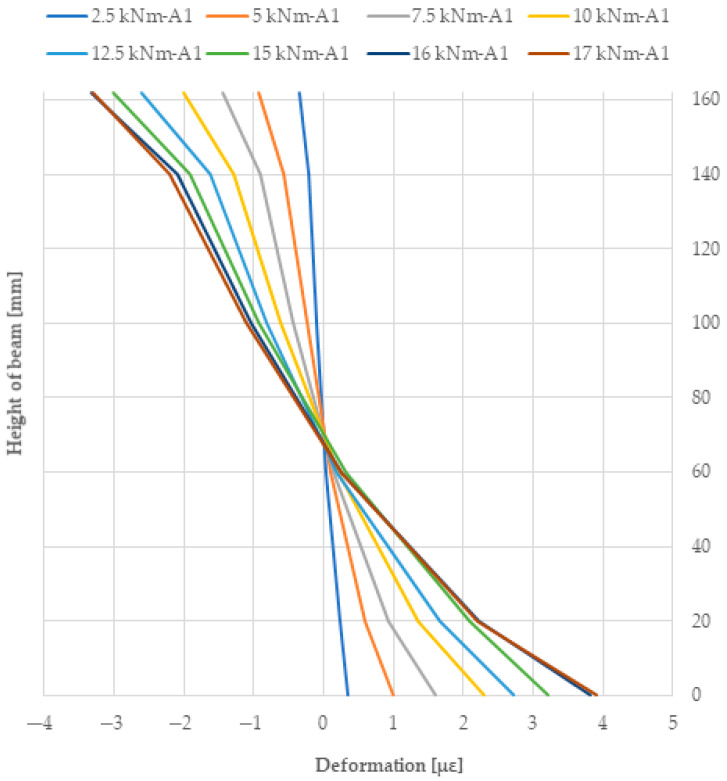
Deformations at the height of the A1 laminated beam at mid-span.

Figure 10 shows the strain plot at the height of the A1 beam section. In contrast, the deformation of the applied reinforcement using pre-stressed basalt, glass, and jute bars was 222.76 MPa, 232.04 MPa and 231.15 MPa, respectively, for an F/2 force of 15 kN. The deformations of the pre-stressed glass bars were significantly higher than those of the basalt bars. In contrast, for beams reinforced with pre-stressed basalt bars, glass bars, and jute bars, the maximum tensile stresses were 51.93 MPa, 59.79 MPa, and 58.71 MPa, respectively, for the 15 kN force. Measuring bases were fixed along the length of the beams and along the length of the pre-stressed bars. The deformed surface of the sandwich-glued beams was therefore tested using extensometers, which were distributed along the timber and composite.

### 3.3. Beam Destruction Analysis

**Figure 11:**
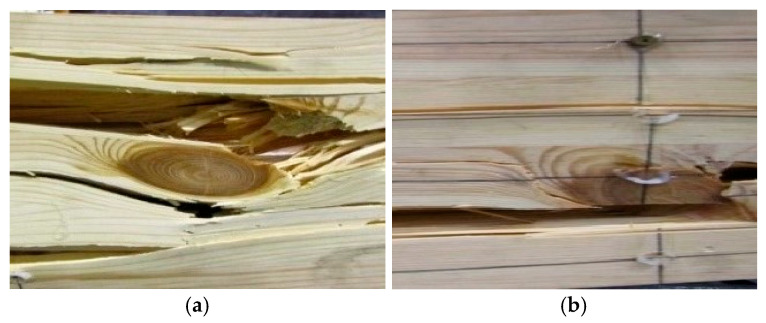
Cracks occurring in the area of knots: (**a**) beam A2 and (**b**) beam A3.

**Figure 12:**
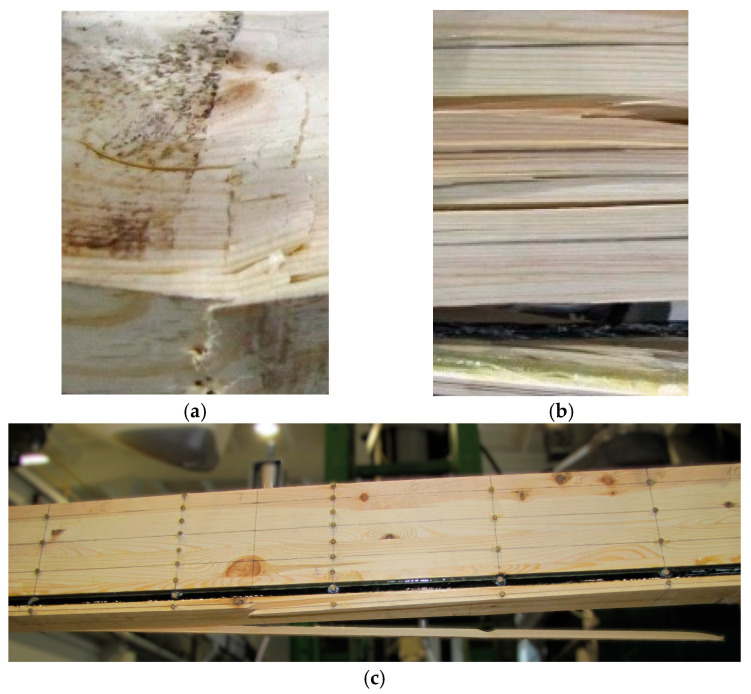
Exemplary images of damage occurring: (**a**) in beam B3, there was crushing of the wood in the compression zone due to a hidden wood defect (e.g., a knot); (**b**) in beam B1, there was fiber shear; (**c**) initial detachment of wood fibers in beam B2 is also shown.

Example images of the failure of wooden beams are shown in Figure 11 and Figure 12. It should be noted that as a result of pre-stressing, the bars worked along the entire length of the beams; this is important in the working of the structure and provides an effective contribution to the strengthening. If no pre-stressing had been applied, then only the epoxy glue would have worked; this would not have provided such an effective bar–wood connection. The work of the bars in the middle of the span also played a very important role in increasing the bending and shear strength of the beams. In beam A1, significant cracking and spalling of parts of the lamellae also occurred in the tension zone close to the point of loading. In beams B1 and B3, fiber shear was also observed, and in beam B3, crushing of the wood of the compression zone occurred due to a hidden wood defect (e.g., a knot) (see Figure 12). In all beams, small fiber cracks appeared. Even in sample S2, there were significant fiber cracks followed by splitting. Largely in beam S1 and S2, there was shearing; this was followed by significant cracking near the support in beam S1, leading to crushing of the timber. In beam J3, small cracks occurred in the tension area followed by fiber spalling. In beams J1 and J3, there was crushing of the compression and tension zones and parallel shear between the support and the load point of the tension zone; furthermore, the existing longitudinal cracks widened further.

### 3.4. Numerical Models

**Figure 13:**
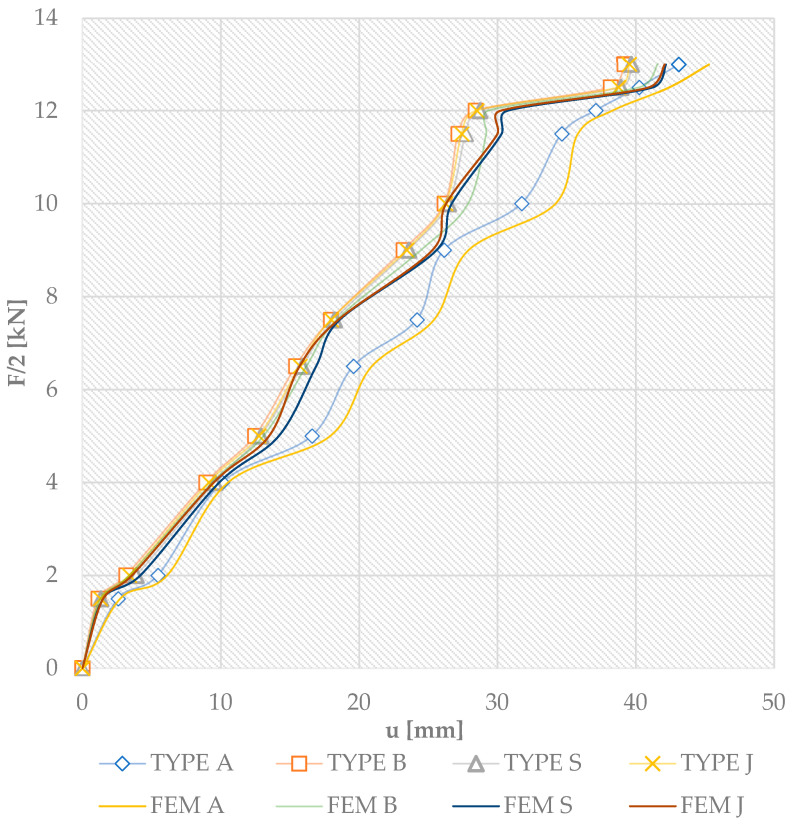
Comparison of the results of the “load–deflection” relationship for all experimentally tested beams with the numerical results [u—deflection].

Figure 13 shows the load–bending relationship within the experimental results and numerical results. As shown below in the figure, the stiffness values of the numerical model are very close to the experimental results.

**Figure 14:**
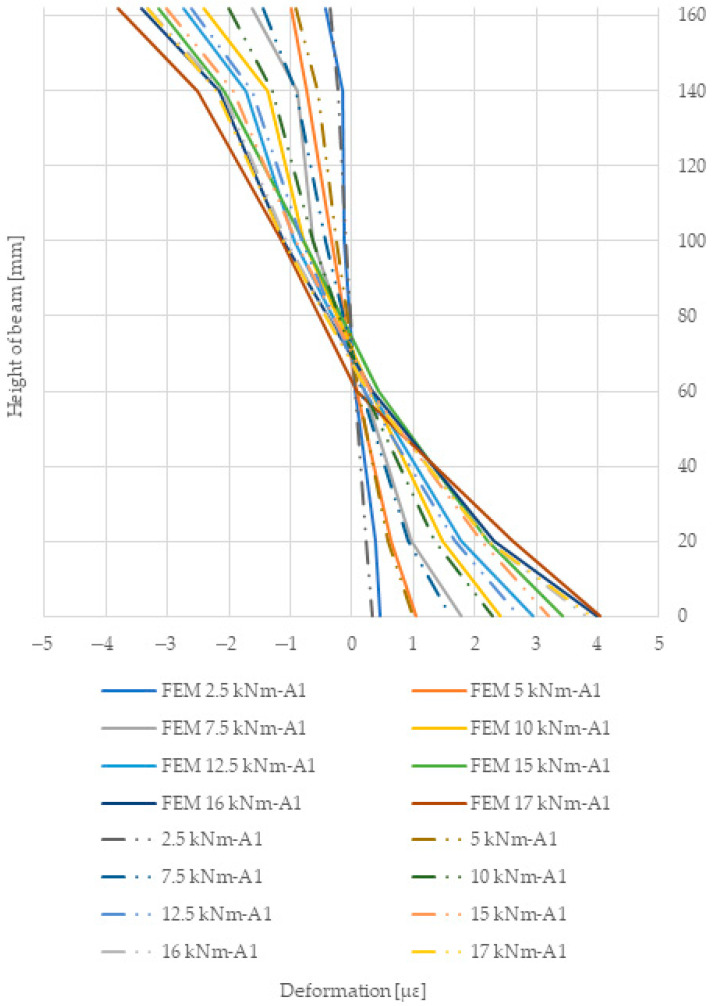
A comparison of tensile and compressive deformations over the height of an A1 laminated beam at mid-span based on experimental and numerical analyses.

Based on the use of reinforcement with pre-stressed basalt bars, the stiffness of the numerical model was approximately 6% higher than for the beams obtained during the experimental tests. In contrast, this difference for pre-stressed beams glued with glass rods was 6.3%. For pre-stressed jute bars, it was 6.5%. Figure 14 below compares the deformation results obtained from the experimental and numerical tests. The difference for compressive deformation is 8.6% and for tensile deformation is 1.8%. It can be said that the results were consistent, although some beams showed slight discrepancies, as described below; these are due to the imperfections of the experimental beams and the present wood defects (see Table 9).

The differences between the numerical and experimental results were primarily caused by inadequacies present in the experimental studies and the idealized, error-free numerical model obtained. Alternatively, this could be the result of the simplifications applied during the numerical analysis. This is because wood is a complex organic material that exhibits anisotropy of mechanical properties. It is also impossible to include all the structural complexities of wood (e.g., irregularities, wood defects, and imperfections) in numerical modelling due to existing limitations. Thus, results obtained from the numerical analysis show high agreement with the experimental results when the materials used are defined by the actual values obtained from experimental tests suitable for each class of wood quality, as determined by the Poisson’s ratios.

## 4. Conclusions

The following conclusions were obtained on the basis of experimental, theoretical, and numerical investigations of “wood–composite” reinforcement in wood beams subjected to bending and shear using natural and artificial fibers.

1. The use of pre-stressed artificial and natural bars to strengthen glued laminated timber beams can effectively increase the load-bearing capacity or stiffness of members in both existing and newly designed structures. It should be noted that the most effective “wood–composite” reinforcement was achieved using BFRP bars, while the static performance analysis of reinforced beams with jute bars produced similar results.
2. A strong bond was achieved at the ‘wood–composite’ joint between the bar and the timber due to correct anchoring, pre-compression, and the presence of matting and roving in the anchorage zone. The anchoring of the bars demonstrated the reinforced beam element’s good working integrity. The 2 mm thick epoxy layer in the contact zone between the reinforcement and the timber was of high quality. Furthermore, no premature delamination was observed between the FRP composite and the wood before the wood failed. To compare different types of reinforcement, the same anchorage and 15 MPa pre-stressing were used for all the reinforced beams, which included plates, 5 mm thick steel sheets, and nuts.
3. The most common form of failure in unreinforced beams was knot cracking in the tension zone. In the case of beams reinforced with pre-stressed bars, crushing of the compression zone and shear along the timber fibers usually occurred initially. The composite bars limited crack propagation.
4. The numerical model closely resembles the experimental model, providing an opportunity to predict the mechanical properties of reinforced beams of different dimensions or materials. This approach can be applied to the design of different reinforcement schemes, particularly with regard to the configuration of wood quality classes. It should be noted that developing and analyzing numerical models will enable structures and applied parameters to be optimized using ‘wood–composite’ materials.
5. This paper [46] describes an experimental research programme which involved strengthening low-quality glued laminated timber beams in bending using glued-in FRP basalt rods. With a small percentage of reinforcement (1.4%) arranged in circular grooves, an average stiffness increase of 8.4% and 10.3% was achieved in global and local measurements, respectively, as well as an average improvement of 23% in load-bearing capacity compared to unreinforced glued laminated timber beams. In these tests, the increase in the load-bearing capacity and stiffness of beams reinforced with pre-stressed basalt bars was approximately 17% and 8%, respectively, compared to unreinforced beams. Further bending tests were performed on creosote-impregnated Douglas fir beams reinforced with glass fiber-reinforced polymer (GFRP) rods, with a reinforcement percentage ranging from 0.27% to 0.82% [47]. The bending strength increased by between 18% and 46% after the tests. However, in this study, the use of pre-stressed glass rods with an 82 × 162 mm beam cross-section resulted in an increase in load-bearing capacity of only around 6%.
6. The application of the above design and material solutions demonstrated the advantages and disadvantages of strengthening the tested wooden elements. This can be achieved without dismantling the internal part of the wooden structure. The analysed strengthening technology is easy and quick to implement when strengthening historic sections of wooden structures. This is achieved by reducing the cross-sectional dimensions of beams made of new wood and using the lowest grades of timber to increase their load-bearing capacity and stiffness.

## Figures and Tables

**Table 1:** Visual sorting, USM, and USC according to the standard PN-D-94021:2013-10 [40], (USM—marginal zone knottiness index, USC—general knottiness index).

Layer	Parameter	Average Value
Lamella 1 KS	USM USC	0.40 0.17
Lamella 4 KS	USM USC	0.45 0.22
Lamella 3 KG	USM USC	0.58 0.30
Lamella 2 KG	USM USC	0.58 0.30

**Table 2:** Material properties of T9 structural lumber (T9 in accordance with PN-EN 14080:2013-07 [42]).

Parameter	Average Value	Standard Deviation
Bending strength [MPa]	19.17	4.11
Compressive strength parallel to fiber [MPa]	16.56	2.07
Modulus of elasticity on bending [MPa]	8150	867
Density [kg/m^3^]	362	-
Moisture content [%]	10.15	0.3

**Table 3:** Material properties of T14 structural lumber (T14 in accordance with PN-EN 14080:2013-07 [42]).

Parameter	Average Value	Standard Deviation
Bending strength [MPa]	33.18	6.82
Compressive strength parallel to fiber [MPa]	29.22	4.35
Modulus of elasticity on bending [MPa]	12,420	920
Density [kg/m^3^]	424	-
Moisture content [%]	12.72	0.7

**Table 4:** Mechanical properties of the laminated timber lamellas used, (KS—medium quality class, KG—lower-quality class).

Layer	Density [kg/m^3^]	Bending Strength [MPa]	Compressive Strength [MPa]
Lamella 1 (KS—medium-quality class)	672.19	63.25	43.21
Lamella 4 (KS—medium-quality class)	654.91	59.16	36.11
Lamella 3 (KG—lower-quality class)	499.78	32.41	29.75
Lamella 2 (KG—lower-quality class)	513.22	34.17	32.08

**Table 5:** Material properties of S&P Resin 55 HP epoxy adhesive [43].

Parameter	Average Value	Standard Deviation
Modulus of elasticity in tension [MPa]	3450	412
Compressive strength [MPa]	106	9.43
Elongation at break (%) acc. to manufacturer	1.73	-

**Table 6:** Material properties of basalt, glass, and jute rods.

Parameter	Average Value Basalt	Standard Deviation	Average Value Glass	Standard Deviation	Average Value Jute	Standard Deviation
Tensile modulus of elasticity [GPa]	81.21	7.21	59.56	7.46	25.9	3.52
Tensile strength [N/mm^2^]	1472	312	1289	287	701	194

**Table 7:** Parameters of glued laminated timber beams.

Type of Beam	Type of Reinforcement
A1	unreinforced
A2	unreinforced
A3	unreinforced
B1	basalt
B2	basalt
B3	basalt
S1	glass
S2	glass
S3	glass
J1	jute
J2	jute
J3	jute

**Table 8:** Mechanical properties of wood and FRP (E_1_, E_2_, E_3_—elastic modulus; v_12_, v_13_, v_23_—Poisson’s ratio; G_12_, G_13_, G_23_—shear modulus).

Parameter	Wood KS	Wood KG	Basalt	Glass	Jute
E_1_ [MPa]	12,420	8150	81.21	59.56	25.9
E_2_ [MPa]	887	582	5.80	4.25	1.85
E_3_ [MPa]	410	269	2.69	1.97	0.86
v_12_	0.54	0.54	0.19	0.19	0.3
v_13_	0.54	0.54	0.19	0.19	0.3
v_23_	0.027	0.027	0.0095	0.0095	0.015
G_12_ [MPa]	678	491	-	-	-
G_13_ [MPa]	678	491	-	-	-
G_23_ [MPa]	67.84	49.13	-	-	-

**Table 9:** Comparison of experimental and numerical results in the tensile zone.

Beam Type	Normal Stresses in Wood, Lamella 1, 16.5 kNm Experimental Model (MPa)	Normal Stresses in Wood, Lamella 1, 16.5 kNm Numerical Model (MPa)	Deflection, 13 kNm Experimental Model (MPa)	Deflection, 13 kNm Numerical Model (MPa)
A	49.68	50.60	43.12	45.3
Standard deviation	4.37	1.22	3.98	1.86
B	52.47	53.78	39.18	41.6
Standard deviation	5.29	2.71	3.67	1.28
S	63.92	65.11	39.69	42.2
Standard deviation	4.61	1.91	4.33	0.78
J	62.12	63.30	39.54	42.1
Standard deviation	5.11	1.35	4.64	0.69

## Data Availability

The original contributions presented in this study are included in the article. Further inquiries can be directed to the corresponding authors.

## Abbreviations

The following abbreviations are used in this manuscript:

FRP	Fiber-reinforced polymer
BFRP	Basalt fiber-reinforced polymer
GFRP	Glass fiber-reinforced polymer
NFRPs	Natural fiber-reinforced plastic composites
CFRP	Carbon fiber-reinforced polymer
FEM	Finite element method
GLULAM	Glued laminated timber
LVL	Laminated veneer lumber
CLT	Cross-laminated timber
NSMR	Near surface-mounted reinforcement
EDSTC	Elliptical FRP–concrete–steel double-skin tubular column
USC	Overall knottiness index
USM	Marginal zone knottiness index
KS	Medium-quality structural lumber class
KG	A class of lower-quality structural lumber
